# Exposure to Microplastics Made of Plasmix-Based Materials at Low Amounts Did Not Induce Adverse Effects on the Earthworm *Eisenia foetida*

**DOI:** 10.3390/toxics12040300

**Published:** 2024-04-18

**Authors:** Beatrice De Felice, Stefano Gazzotti, Maddalena Roncoli, Eleonora Conterosito, Valentina Gianotti, Marco Aldo Ortenzi, Marco Parolini

**Affiliations:** 1 Department of Environmental Science and Policy, University of Milan, Via Celoria 26, I-20133 Milan, Italy; beatrice.defelice@unimi.it; 2 Department of Chemistry, University of Milan, Via Golgi 19, I-20133 Milan, Italy; stefano.gazzotti@unimi.it (S.G.); marco.ortenzi@unimi.it (M.A.O.); 3 Department of Sustainable Development and Ecological Transition, University of Piemonte Orientale, Via T. Michel 11, I-13100 Vercelli, Italy; maddalena.roncoli@uniupo.it (M.R.); eleonora.conterosito@uniupo.it (E.C.); valentina.gianotti@uniupo.it (V.G.)

**Keywords:** biomarker, non-recyclable plastics, soil, terrestrial organisms

## Abstract

The implementation of recycling techniques represents a potential solution to the plastic pollution issue. To date, only a limited number of plastic polymers can be efficiently recycled. In the Italian plastic waste stream, the residual, non-homogeneous fraction is called ‘Plasmix’ and is intended for low-value uses. However, Plasmix can be used to create new materials through mechanical recycling, which need to be tested for their eco-safety. This study aimed to investigate the potential toxicity of two amounts (0.1% and 1% MPs in soil weight) of microplastics (MPs) made of naïve and additivated Plasmix-based materials (Px and APx, respectively) on the earthworm *Eisenia foetida*. Changes in oxidative status and oxidative damage, survival, gross growth rate and reproductive output were considered as endpoints. Although earthworms ingested both MP types, earthworms did not suffer an oxidative stress condition or growth and reproductive impairments. The results suggested that exposure to low amounts of both MPs can be considered as safe for earthworms. However, further studies testing a higher amount or longer exposure time on different model species are necessary to complete the environmental risk assessment of these new materials.

## 1. Introduction

Since the 1950s, plastics have revolutionized our lives, providing us with materials used in all production sectors and in everyday life. Their peculiar features, including lightness, resistance, versatility and low production cost, have allowed the use of plastics in diverse applications, including packaging (39%); building and construction (23%); automotive (8%); electrical and electronics (6%); agriculture, farming and gardening (4%); and houseware, leisure and sports (4%) [1]. Thus, global annual plastic production continues to increase unabated, reaching 440.3 Mt in 2022 [1]. However, the inappropriate management and disposal of waste at the end of life of plastic objects caused their massive accumulation in the environment, resulting in worrisome contamination of both aquatic and terrestrial ecosystems worldwide [2,3,4]. A plethora of different plastic items have been ubiquitously detected in both abiotic and biotic environmental matrices collected in anthropic and natural ecosystems, giving plastic the role of the main contributor to the contamination fingerprint of the Anthropocene, also defined as the ‘plastic age’ [5]. To reduce the loss and the environmental impact of plastic waste, the implementation and promotion of recycling techniques, as well as the transition to biodegradable plastics, have been identified as valid strategies [6,7]. To date, the current European target for plastic recycling is 22.5%, and a future goal is 32.5% of plastic waste production [8], although some member states are approaching the 2025 goal [9]. Despite these promising trends, increasing the recycling rate is difficult because the waste streams from plastic packaging collected in recovery facilities are very heterogeneous and difficult to separate [10]. In Europe, Italy ranks as the second-largest plastic consumer and generates over 1 million tons of plastic waste per year, but only half of this amount is effectively recycled. In fact, at present, only packaging made of polyethylene terephthalate (PET), polyethylene (PE), and polypropylene (PP) is efficiently sorted and profitably regenerated [11]. In contrast, the remaining plastics are made of unrecycled and unrecyclable materials because of their excessive heterogeneity or multi-layer composition (plastics, cardboard, aluminum or other components) [12]. In the Italian plastic waste stream, this residual, non-homogeneous fraction is called ‘Plasmix’ [11]. Specifically, Plasmix consists of plastics (57%), paper and cardboard (10%), wood (3%), textiles (3%), inert and others (27%) [13]. Among plastics, the main contributor is represented by polyolefin mixture (i.e., PE and PP; 60–70%), followed by PET (4–5%), PS (2–4%) and low amounts of polyamides (PAs), acrylonitrile butadiene styrene (ABS), expanded polystyrene (XPS) and polyurethanes (PU) or other polymers [11]. Because of its non-homogeneous and variable composition, the separation of different polymer fractions constituting Plasmix is very difficult and economically not advantageous. Thus, Plasmix is mainly incinerated (57%), used as a substitute for coal burning in cement kilns (27%) or landfilled (16%) [11]. Considering the increase in the complexity of plastic packaging and the efficiency of the recycling process, the amount of non-recyclable plastics is constantly increasing. For this reason, there is an urgent need to develop strategies for managing and valorizing Plasmix, but to date, only a few pioneering industrial applications have been implemented to exploit this waste. For instance, Plasmix has been used by VGM Patent (Italy) to create adhesives for wood shavings, by Revet Recycling and R3direct (Italy) to produce 3D printing filaments and by Tesco (UK) as additives for asphalts. Moreover, in 2019, Consorzio Nazionale per la Raccolta, il Riciclo e il Recupero degli Imballaggi in Plastica (COREPLA) and Ente Nazionale Idrocarburi (ENI) activated a trial to develop a process for producing hydrogen from Plasmix [6]. Recently, a novel approach for reusing Plasmix has been implemented; through an advanced mechanical recycling process, Plasmix was added with a compatibilizer additive to create a more homogeneous material virtually behaving as the combination of its components [14]. New Plasmix-based materials have been examined by eco-designers, who proposed possible objects and markets for valorizing Plasmix as secondary material [15]. However, before being used in object production, the new Plasmix-based (plastic) material needs to be tested for investigating potential environmental risks and toxicity towards organisms. Ecotoxicology provides valuable tools for assessing the risk related to materials and selecting eco-friendly and sustainable ones [16]. The application of standardized and/or novel ecotoxicological tests integrates the characterization of materials allowing the identification of potential targets and mechanisms of toxic action in model species at different levels of the bio-ecological hierarchy [16]. This ecotoxicological evaluation is particularly important in the assessment of new plastic materials. Indeed, once in the environment, plastic materials might suffer degradation and/or fragmentation due to weathering processes, leading to the formation of microplastics (MPs; i.e., any plastic item in the 1 to <1000 µm size range—[17]) and/or the release of additives/chemicals associated to the material. Several studies have shed light on the diverse adverse effects induced by the exposure to MPs made of different polymers and additives towards aquatic [18,19] and terrestrial organisms [20]. Focusing on terrestrial organisms, most of the studies were performed on earthworms, showing that the exposure to different and very high levels of MPs made of different polymer compositions, sizes and shapes did not induce effects on the survival, reproduction and growth but triggered the disruption of redox homeostasis, the onset of oxidative stress and histological damage [20]. However, as the reuse of Plasmix as a secondary raw material is very limited; to date, no studies have investigated the potential toxicity of MPs originating from a Plasmix-based material towards any model organisms.

Thus, the present study aimed at investigating and comparing potential adverse effects at biochemical and individual levels induced by the exposure to two amounts (0.1% and 1% MPs in soil weight) of MPs made of Plasmix-based materials towards the earthworm *Eisenia foetida*. A battery of oxidative stress biomarkers, including the activities of antioxidant (SOD, CAT and GPx) and detoxifying (GST) enzymes and lipid peroxidation, was applied to explore alterations at the sub-individual level. Changes in survival, gross growth rate and reproductive output were investigated as effects at the individual level.

## 2. Materials and Methods

### 2.1. Preparation of Microplastics from Plasmix-Based Materials

In order to test the potential toxicity of Plasmix, Plasmix-based materials were created according to the procedures described in detail elsewhere [14] and then micronized to obtain microplastics (MPs), which were then administered to earthworms. A brief description of the procedures used for preparing Plasmix-based materials and related MPs is reported in the further paragraphs.

#### 2.1.1. Pre-Treatment of Waste

Waste samples were provided by Consorzio Nazionale per la Raccolta, il Riciclaggio e il Recupero degli Imballaggi in Plastica (COREPLA). Wastes were sorted by hand, and all the non-plastic fractions were removed from the mixture. The complex mixture of plastic wastes was washed with tap water and dishwashing detergent through multiple cycles and finally rinsed with tap water. The washed plastic mixture was chopped into smaller pieces through a series of freezing (in liquid nitrogen) and grinding cycles, according to the procedure previously used to obtain MP from different plastic materials [21,22].

#### 2.1.2. Extrusion of Plasmix

The pre-treated, chopped Plasmix mixture reflected the typical composition of Plasmix from the Italian plastic waste stream [11]. It was directly added to the feeder of a twin-screw lab-scale extruder. The extrusion was carried out at a temperature ranging between 200 °C at the feeding and 240 °C in the central zone, with twin screws rotating at 210 rpm. The extruded material was cooled at room temperature, and it was ground into small granules with a laboratory grinder. Melt extrusions were performed with a Thermo Scientific Process 11 double-screw extruder with a screw diameter of 11 mm and L/D = 40.

#### 2.1.3. Additivation of Plasmix

Before the extrusion, a dry blend of about 200 g of pre-treated, chopped Plasmix with 4% wt. of Vibatan peroxide was mixed in a 500 mL beaker for a preliminary homogenization. The dry blend obtained was then directly added to the feeder of a twin-screw lab-scale extruder. The extrusion was carried out at a temperature ranging between 220 °C at the feeding and 260 °C in the central zone, with twin screws rotating at 100 rpm. The extruded material was cooled at room temperature, and it was ground into small granules with a laboratory grinder. Melt extrusions were performed with a Thermo Scientific Process 11 double-screw extruder with a screw diameter of 11 mm and L/D = 40.

#### 2.1.4. Injection Molding

The granules obtained from the extrusion process were processed through injection molding. First, 150 g of pure Plasmix granules were added directly to the feeder. The injection molding process was performed at a temperature ranging between 180 °C and 190 °C and pressure between 35 and 37 bar, to yield dogbone specimens (ISO 527-5A) [23]. Injection molding was conducted with a Babyplast injection molding machine. The same processing conditions were applied to both the additivated and pristine Plasmix samples. The dogbone specimens made of Plasmix-based and additivated Plasmix-based material were used to create MPs. The dogbone specimens made of Plasmix-based and additivated Plasmix-based materials were brownish/army green in color.

#### 2.1.5. Preparation of Microplastics from Plasmix-Based Materials

**Figure 1:**
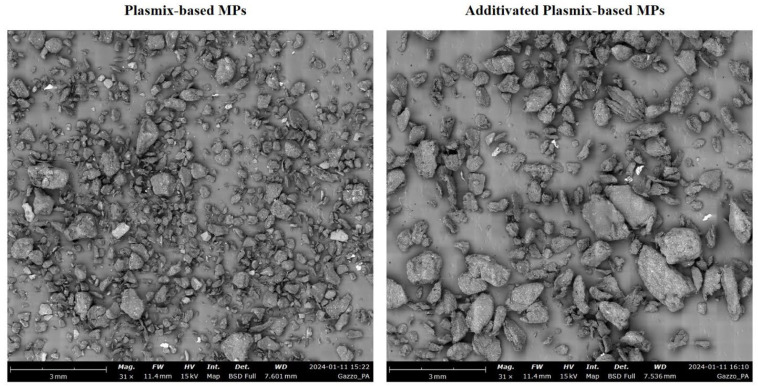
Images of Plasmix-based microplastics (**left** panel) and additivated Plasmix-based microplastics (**right** panel).

The dogbone specimens made of Plasmix-based and additivated Plasmix-based materials underwent a series of freezing (in liquid nitrogen) and grinding cycles, according to the procedure previously used to obtain MPs from different plastic materials [21,22]. At the end of this procedure, the items were sorted using a 1 mm sieve to select only particles in the range of MPs (1 µm–1 mm; [17]). MPs originating from the grinding of Plasmix-based (hereafter Px-MPs) and additivated Plasmix-based (hereafter APx-MPs) materials had an irregular shape and variable size (Figure 1). Spectra of Px-MPs and APx-MPs were obtained using a Fourier transform infrared spectroscope (FT-IR; Perkin Elmer Spectrum 100, Waltham, MA, USA). Pictures of Px-MPs and APx-MPs (Figure 1) were captured under a scanning electron microscope (LEO1430, Zeiss, Oberkochen, Germany).

The perimeter, area, diameter and circularity (i.e., 4π × area/perimeter^2^) of 573 Px-MPs and 440 APx-MPs were measured using ImageJ freeware software (Version 1.54i). Considering the irregularity of the items, their size was calculated as the diameter of a spherical particle having the same area.

### 2.2. Gas Chromatographic Characterization (HS-GC-MS) of Px-MPs and APx-MPs

About 0.50 mg of Px-MPs or APx-MPs was introduced into a 10 mL pre-weighed headspace glass vial and immediately closed with a pierceable PTFE/silicone septum cap. Each vial was heated by a hand-made instrument in an oil bath for 1 h at 120 °C. Ten minutes before the end of the heating time, the septum of the cap was pierced with the sampling syringe (gas-tight glass syringe) to heat and condition the syringe itself to avoid condensation phenomena. Then, 3 μL of headspace gases were injected for the GC-MS analysis. In every chromatogram, the peak area (Apeak) of every peak was registered and scaled for the sample weight (hweight) to obtain a comparable area value (SApeak) All the materials were tested in triplicate. The average values of H calculated as reported above, consequently, represent semi-quantitative and comparable results among the experiments. Finnigan Trace GC Ultra and Trace DSQ were used for the GC-MS analysis, employing a Phenomenex ZB-WAX MS capillary column (30 m, 0.25 mm i.d., 0.25 μm thickness), a 250 °C injector temperature, the splitless mode of operation, and helium as carrier gas at 1.0 mL min^−1^. The MS transfer line and oven temperatures were set at 300 °C and 150 °C, respectively. The column heating gradient consisted of the steps reported in Table 1, which allowed obtaining a chromatographic run of 44 min.

The MS signal was acquired in EI+ mode with an ionization energy of 70.0 eV, and the ion source temperature of 290 °C was employed. The acquisition was performed in full scan mode in the 35–500 *m*/*z* range.

### 2.3. Experimental Plan

*Eisenia foetida* earthworms were purchased from the Consorzio Italiano Allevamento Lombrichi (Con.It.A.Lo.). Earthworms of the same age class (3 months old) were maintained in large glass vessels filled with 3 kg of natural soil for 7 days for acclimation to our laboratory conditions, i.e., temperature 20 ± 1 °C, humidity close to 80%, pH = 6.5. Soil was collected in a small, private vegetal garden (coordinates: 45.794755; 8.255989) in a small hamlet (<100 inhabitants; 604 m a.s.l.) of Varallo, a municipality located in Northern Piedmont (Italy). A preliminary analysis of soil biodiversity confirmed the lack of contamination, the presence of earthworms and, consequently, the soil’s suitability for maintenance and exposure of earthworms under laboratory conditions.

Before the inclusion of earthworms, the soil was sieved (<1 mm) to remove coarse fragments and residuals of grass and roots. The texture (clay: <0.002 mm = 9.38%; silt: 0.002–0.05 mm = 30.74%; sand: 0.05–2 mm = 59.68%; gravel: >2 mm = 0.2%), pH (6.4), carbonate content (<1 g/Kg dry soil), organic carbon content (75.8 g/Kg dry soil) and total organic matter content (131 g/Kg dry soil) of soil used in this experiments were certified by the Minoprio Analisi e Certificazioni S.r.l. laboratory (Vertemate con Minoprio (CO), Italy).

Five experimental groups were planned (i.e., control, 0.1 and 1% Px-MPs in soil weight, and 0.1 and 1% APx-MPs in soil weight). At the end of the acclimation period, 15 earthworms per experimental group were individually stocked in 50 mL glass beakers filled with 40 g of soil and exposed for 14 days to two amounts of Px-MPs and APx-MPs (0.1 and 1% Px-MPs or APx-MPs in soil weight) (Experiment 1). These amounts were similar to the lowest ones administered to earthworms in some previous studies testing the potential toxicity of different MPs [24,25,26,27]. The mean (±standard deviation) body weight of earthworms assigned to each experimental group was 1.78 ± 0.26 g for the control group, 1.37 ± 0.19 g and 1.45 ± 0.4 g for 0.1 and 1% Px-MPs, and 1.41 ± 0.27 g and 1.49 ± 0.20 g for 0.1 and 1% APx-MPs in soil weight, respectively. Earthworms’ body weight did not significantly differ between the experimental groups (Student’s *t*-test, *p* > 0.05 for all the pairwise comparisons). The exposure lasted 14 days, and it was performed under static, non-renewal conditions according to Test No. 207: Earthworm, Acute Toxicity Tests [28]. Experiments were carried out in a thermostatic chamber (temperature = 20 °C) with humidity close to 80% and under a 16:8 h light/dark photoperiod. To obtain 0.1 and 1% MPs in soil weight, 0.4 g or 4 g of Px-MPs or APx-MPs, respectively, was added to the soil, which was mixed using a stainless-steel shovel to ensure the MPs were homogeneously distributed into the soil. The amount of administered MPs was assessed by counting their number in 1 g of soil after the homogenization procedure (*n* = 3 replicates per experimental group). In the 0.1 and 1% Px-MP experimental groups, we counted a mean (± standard deviation) of 32.66 ± 3.05 and 134.00 ± 9.84 MPs per g of soil, respectively. In the 0.1% and 1% APx-MP experimental groups, 52.66 ± 8.62 and 143.33 ± 7.76 per g of soil were counted, respectively. Each beaker (i.e., replicate) was covered with tinfoil to avoid airborne MP contamination and the escape of earthworms. Every day, the survival of the earthworms was checked.

At the end of the exposure, mortality was recorded, and body weight (±0.01 g) of all the alive individuals was measured after washing and drying with paper towels. Body weight was then used to calculate the gross growth rate (gGR) of each individual according to [29]. The gross growth rate was considered as a proxy of the growth of earthworms, assuming that individuals with similar body weights have similar amounts of gut content and, therefore, exhibit a similar ratio of gut content to body weight [29]. After weighing, the anterior part (up to the clitellum) of ten individuals was cut, frozen in liquid nitrogen and maintained at −80 °C until biochemical analyses (Section 2.4). Five whole earthworms for each condition were fixed in 96% ethanol for 24 h. Dissection was performed to check for the presence of Px-MPs or APx-MPs in earthworms’ digestive tracts through optical microscopy (Leica EZ W).

An additional experiment (*Experiment 2*) was performed to assess reproductive impairments due to exposure to Px-MPs or APx-MPs. Ten adult earthworms were placed in 3 L glass containers filled with 1 Kg of soil and exposed for 28 days to the same amounts, under the same experimental conditions, described above. According to [30], at the end of the exposure, earthworms were removed from the container, and the number of cocoons was counted according to [31].

### 2.4. Oxidative Stress Biomarkers

The anterior part (up to the clitellum) of two earthworms (~0.3 g per individual) was pooled and homogenized with a motor pestle in 100 mM potassium phosphate buffer, supplemented with KCl (100 mM), EDTA (1 mM), dithiothreitol (DTT; 1 mM) and protease inhibitors (1:100 *v*/*v*) at pH = 7.4. Five pools per experimental group were prepared. Before centrifugation, 200 µL of the raw homogenate was taken from each sample and stored at −80 °C for the lipid peroxidation analyses. The homogenates were centrifuged at 17,000× *g* for 30 min, and the supernatant was processed to measure protein content and enzyme activity. The spectrophotometric methods for the determination of protein content and oxidative stress biomarkers were described elsewhere [27].

Briefly, protein content was determined according to the Bradford method [32]. The superoxide dismutase (SOD) activity was measured by determining the inhibition of the reduction of cytochrome c (10 µM) caused by superoxide anion generated through the xanthine oxidase (1.87 mU/mL)/hypoxanthine (50 mM) reaction for 1 min at λ = 550 nm. The catalase (CAT) activity was evaluated by measuring the consumption of H_2_O_2_ (50 mM) for 1 min at λ = 240 nm. The glutathione peroxidase (GPx) activity was evaluated through the measurement of the NADPH consumption (0.12 mM) in a buffer supplemented with glutathione (2 mM), sodium azide (1 mM) and glutathione reductase (2 U/mL), using H_2_O_2_ (0.2 mM) as a substrate, for 1 min at λ = 340 nm. The glutathione S-transferase (GST) activity was measured for 1 min at λ = 340 nm, using reduced glutathione (1 mM) and 1-chloro-2,4 dinitrobenzene (CDNB; 1 mM) as substrates. Levels of lipid peroxidation were measured according to the thiobarbituric acid reactive substance (TBARS) method [33]. All the biomarkers were performed using reagents purchased from Merck (Darmstadt, Germany), while the readings were acquired with a Genova Bio spectrophotometer (JenwayTM, Fisher Scientific Italia, Segrate (MI), Italy).

### 2.5. Statistical Analysis

The effects of the amount (0.1% and 1% MPs in soil weight), the type of MPs (Px-MPs and APx-MPs) and their interactions on protein content, oxidative stress biomarkers, gross growth rate and reproductive output were investigated through a two-way analysis of variance (ANOVA), after checking for normality and homoscedasticity of data by the application of Shapiro–Wilk and Levene’s tests, respectively. Significant differences among groups (* *p* < 0.05 and ** *p* < 0.01) were checked by means of Tukey’s post hoc test. All the statistical analyses were run in R 4.03 [34] using the *lmer* package.

## 3. Results

### 3.1. Morphometric Features of Px-MPs and APx-MPs

The morphometric features of Px-MPs and APx-MPs are reported in Table 2.

Px-MPs and APx-MPs differed in size and morphometry (Table 2). APx-MPs had significantly different size parameters compared to Px-MPs, having larger area (unpaired Student’s *t*-test; t = 14.563; *p* < 0.001), perimeter (t = 20.137; *p* < 0.001) and diameter (t = 11.092; *p* < 0.001). In contrast, the shape of MPs was similar, approximating a spherical shape, as the circularity of both MP types was close to 0.74 (t = 0.150; *p* = 0.880). Finally, Px-MPs and APx-MPs were grouped into seven size classes based on their diameter (Table 3).

### 3.2. Identification of Contaminants in Px-MPs and APx-MPs

**Figure 2:**
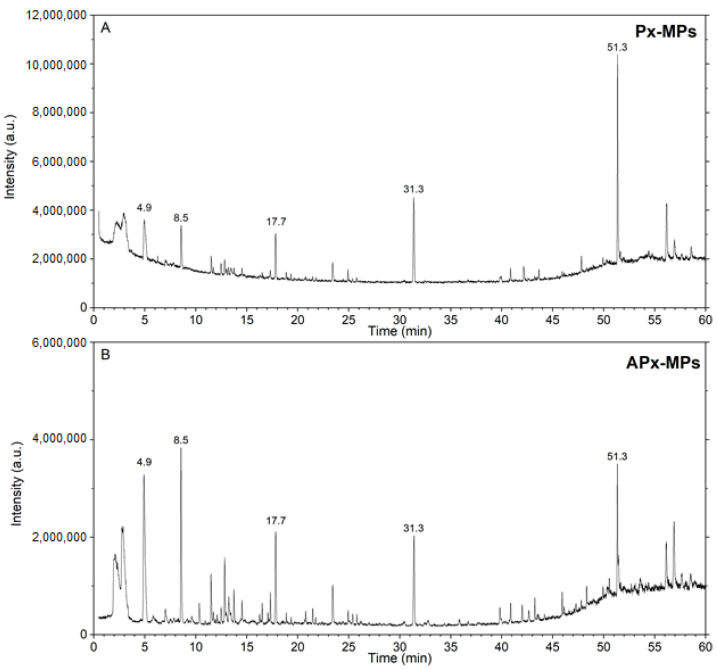
GC-MS chromatogram of PX-MPs (**A**) and APx-MPs (**B**).

Px-MPs and APx-MPs were subjected to analysis by HS-GC-MS. Typical chromatograms are shown in Figure 2. It is important to point out that the gradient conditions used in gas chromatographic separation (Table 4) allow the detection of the most volatile molecules in the first part of the chromatogram, and then the low increase in GC oven temperature also allows the resolution of the high-mass molecules that evolve from the samples.

A good resolution can be observed. The peak identification was performed by the comparison of the mass spectra collected for every peak with the mass spectra contained in the Wiley and NIST libraries. A peak height > 10,000 has been set as a threshold for proceeding with the identification. The results summarized in Table 4 report the name of the identified molecules and the CAS number: 27 peaks were identified.

As expected, the molecules identified were those that typically evolve from plastic-made samples as residual monomers, aliphatic and aromatic hydrocarbons, ketones and carboxylic acid. Their presence, as reported in the literature, may come from two significant contributions. They can be ascribed to the history of the material itself [35], as during their production, processing and use, the different Plasmix constituent materials are subjected to high temperatures under different conditions (e.g., vacuum, nitrogen or air). The second contribution derives from the formation of degradation products due to the conditions of the process employed in the present study to obtain the sample Px-MPs and APx-MPs [36,37]. These conditions result in degradation reactions that generate changes in the properties of the polymers (e.g., reduction in the molecular mass and intrinsic viscosity and yellowing) and the emission of volatile substances including aldehydes, aromatic hydrocarbons and ketones. The two chromatograms displayed in Figure 2 show that no remarkable differences occurred and that the evolution profiles of the molecules were very similar. The difference in the intensity of the different peaks that can be observed does not appear significant in differentiating the added sample (APx-MPs) from the original one. Rather, it accounts for the fact that the material is very non-homogeneous as the same trend was obtained for five genuine replicate analyses performed for both Px-MPs and APx-MPs.

### 3.3. Ingestion of Px-MPs and APx-MPs

**Figure 3:**
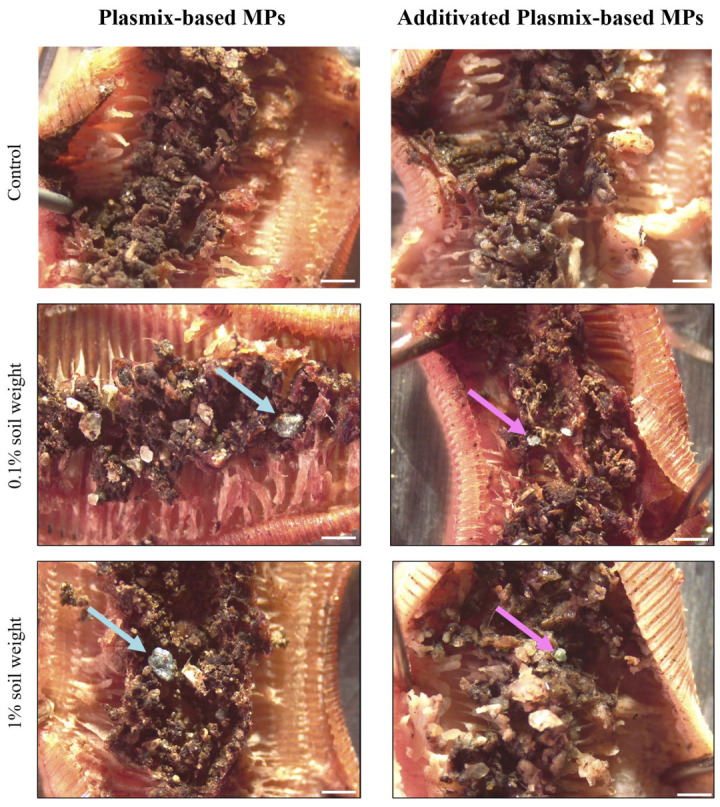
Dissections of the digestive tract of earthworms exposed to 0.1% and 1% soil weight of microplastics (MPs) made of Plasmix-based material (Plasmix-based MPs) or additivated Plasmix-based material (additivated Plasmix-based MPs). Light blue (**left** panels) and violet (**right** panels) arrows show the presence of Plasmix-based MPs and additivated Plasmix-based MPs in earthworms’ digestive tracts, respectively. Scale bar = 1 mm.

Microscopy analyses confirmed that earthworms were able to efficiently ingest both Px-MPs and APx-MPs in all the experimental groups, except for the control group where no MPs were detected in the gut of earthworms (Figure 3).

### 3.4. Effects of Px-MPs and APx-MPs

**Figure 4:**
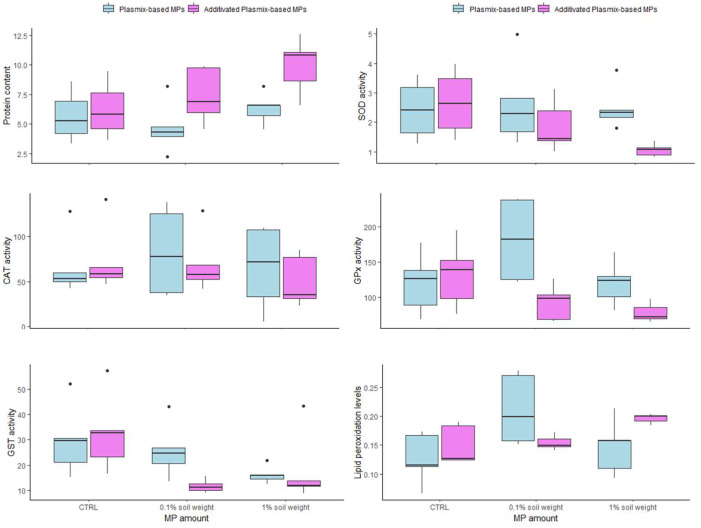
Box plots of protein content, superoxide dismutase (SOD) activity, glutathione peroxidase (GPx) activity, catalase (CAT) activity, glutathione S-transferase activity and lipid peroxidation levels measured in pools of *Eisenia foetida* individuals (n = 5 pools of two individuals each) after 14 days of exposure to two amounts (0.1% and 1% MPs in soil weight) of MPs from Plasmix-based and additivated Plasmix-based materials.

No mortality occurred after 14 days (Experiment 1) or 28 days (Experiment 2) of exposure to both amounts of Px-MPs and APx-MPs. The results of biochemical analyses are reported in Figure 4. The protein content of earthworms exposed to APx-MPs was significantly lower compared to that of conspecifics treated with Px-MPs (F_1,24_ = 8.669; *p* = 0.007) independently of the MP amount tested. Indeed, no effect of MP amount (F_2,24_ = 3.311; *p* = 0.053) or MP amount × MP type interaction was noted (F_2,24_ = 1.335; *p* = 0.282). Although the activity of SOD in earthworms exposed to APx-MPs was significantly lower compared to that of conspecifics treated with Px-MPs (F_1,24_ = 4.486; *p* = 0.044), no effect of MP amount (F_2,24_ = 1.249; *p* = 0.304) or MP amount × MP type interaction was noted (F_2,24_ = 1.456; *p* = 0.252). Similarly, the GPx activity measured in earthworms exposed to APx-MPs was significantly lower compared to that of conspecifics from the Px-MP group (F_1,23_ = 8.410; *p* = 0.008); no effect of MP amount (F_2,23_ = 1.785; *p* = 0.190) or MP amount × MP type interaction (F_2,23_ = 3.080; *p* = 0.065) was found. No significant effect of MP amount (F_2,24_ = 0.580; *p* = 0.567), MP type (F_1,24_ = 0.448; *p* = 0.509) or MP amount × MP type interaction (F_2,24_ = 0.114; *p* = 0.892) was found for CAT activity. Similar results were also obtained for GST activity, with no significant effect of MP type (F_1,24_ = 1.049; *p* = 0.315) or MP amount × MP type interaction (F_2,24_ = 1.519; *p* = 0.239). However, a significant effect of the MP amount on GST was noted (F_2,24_ = 3.868; *p* = 0.034), with levels measured in earthworms exposed to 1% MPs in soil weight lower than those observed in control individuals (*p* = 0.043), independently of the MP type. Lipid peroxidation levels were significantly affected by the MP amount (F_2,22_ = 4.537; *p* = 0.022), with lower levels measured in control individuals compared to those measured in the 0.1% soil weight MP group (*p* = 0.019), independently of MP type. In fact, no effect of MP type (F_2,22_ = 0.206; *p* = 0.653) or MP amount × MP type interaction (F_2,22_ = 3.436; *p* = 0.051) was found.

**Figure 5:**
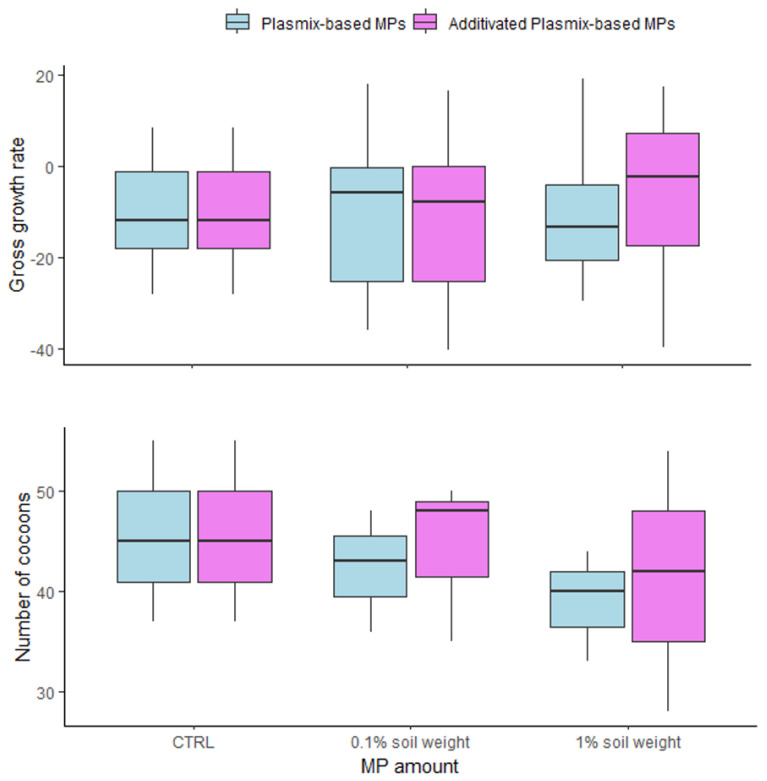
Box plots of gross growth rate (**upper** panel) and reproductive output (**lower** panel) of *Eisenia foetida* individuals after 14 days of exposure to two amounts (0.1% and 1% MPs in soil weight) of MPs from Plasmix-based and additivated Plasmix-based materials.

No significant effect of MP amount (F_2,77_ = 0.101; *p* = 0.903), MP type (F_1,77_ = 0.277; *p* = 0.599) or MP amount × MP type interaction (F_2,77_ = 0.566; *p* = 0.569) was found on gGR. Similarly, neither MP amount (F_2,12_ = 0.589; *p* = 0.570), nor MP type (F_2,12_ = 0.121; *p* = 0.734), nor MP amount × MP type interaction (F_2,12_ = 0.030; *p* = 0.969) affected the reproductive output of earthworms (Figure 5).

## 4. Discussion

Our study demonstrated that the exposure to MPs made of Plasmix-based materials, both native (Px-MPs) and additivated with an industrial peroxide (APx-MPs), were ingested but did not induce acute or chronic toxic effects on earthworms.

Several studies confirmed that different species of earthworms can easily ingest (and egest) MPs displaying a wide range of shapes and sizes made of fossil-based polymers (i.e., conventional plastics) and/or bio-based polymers (i.e., bioplastics). The ingestion of polyester microfibers [26] and MPs made of low-density PE (LDPE; [29,38]), PE and PS [39], polylactic acid (PLA), polypropylene carbonate (PCC; [30]) and polybutylene adipate co-terephthalate (PBAT; [29]) has been demonstrated in earthworms belonging to the *Eisenia* and *Lumbricus* genera. However, some studies suggested that the size of MPs could drive their ingestion, with earthworms preferring small-sized items. For instance, after the administration of LDPE-MPs lower than 400 µm in size, *E. foetida* individuals preferentially ingested items smaller than 100 μm [24]. Similarly, after exposure to PE-MPs of which half had a size lower than 50 μm, 90% of MPs detected in casts of *Lumbricus terrestris* were smaller than 50 μm [38]. Moreover, the polymer composition of MPs and the presence/release of additives could influence their ingestion by earthworms [40]. However, other studies suggested that earthworms ingested MPs regardless of their polymer composition, concluding that they did not prefer ingesting items of a particular polymer [26,41,42]. Although the Px- and APx-MPs administered to earthworms differed in size, they had the same intrinsic polymeric composition. Moreover, the semi-quantitative analyses performed with HS-GC-MS that measured the release via volatilization of chemical compounds from Px-MPs and APx-MPs, simulating the exposure of organisms occurring in soil, showed no differences between plastic types. Although we did not quantify the concentrations of released compounds, the analysis of the chromatograms suggested a very low release from both MP types. However, it is important to consider that such release occurred after heating MPs at 120 °C, which is a more drastic condition compared to the real environment. Thus, we can suppose that the release of chemical compounds in soil from both Px-MPs and APx-MPs should not contribute to inducing toxic effects on earthworms. Despite differences in size and similarity in polymer composition and release of compounds, Px-MPs and APx-MPs were easily ingested by earthworms. In fact, a visual inspection of their digestive tracts confirmed the ingestion of items of all sizes, regardless of their composition (Figure 3).

Despite Px-MPs and APx-MPs being ingested, no acute effect, in terms of changes in survival, was observed in treated earthworms compared to control conspecifics after 14 or 28 days of exposure. These results confirmed previous findings on *E. foetida* or similar earthworm species exposed to MPs having different sizes, shapes and polymeric compositions, originating from both fossil-based plastics and bioplastics [26,29,38,43]. In addition, no alterations at the biochemical level were caused by the exposure to both MP types (Figure 4). Even if lower levels of SOD and GPx were observed in individuals from the APx-MP experimental group compared to the Px-MP one, the activities of both these enzymes did not significantly differ compared to those measured in control individuals. Similarly, the activities of CAT and GST were also not affected by the administered amounts of both MP types. Thus, under our experimental conditions, the exposure to Px-MPs and APx-MPs did not alter the oxidative status of earthworms and, consequently, did not trigger the onset of oxidative stress, as ultimately confirmed by the lack of increase in oxidative damage (i.e., lipid peroxidation levels). Our findings disagreed with those reported in previous studies that tested the capability of MPs to cause oxidative stress in earthworms. Indeed, some studies showed that MP exposure caused oxidative stress in *Eisenia foetida* earthworms through the modulation of antioxidant and detoxifying enzyme activities, as well as the onset of oxidative damage to lipids [44]. For instance, the exposure to high-density PE- (HDPE; 28–400 μm size range) and PP-MPs (8–1660 μm size range) reduced the activities of SOD, CAT and GST and increased DNA oxidation [45]. The exposure to LDPE-MPs (<400 μm in size) increased CAT activity and lipid peroxidation levels only at amounts higher than 1.0 g/Kg [24]. A decrease in SOD activity coupled with an increase in glutathione (GSH) content was induced by a 28-day exposure to 0.1 and 1 mg of 0.1 and 1.3 μm PS-MPs [25]. Moreover, a significant activation of CAT and GPx, followed by the inhibition of SOD and GST, caused an increase in lipid peroxidation after a 14-day exposure to 20% soil weight of PE (≤300 μm) or PS-MPs (≤250 μm) [38]. These studies suggested that only the administration of MP amounts higher than those we administered induced toxic effects on earthworms. Indeed, no significant effects were found on most oxidative stress biomarkers at exposure amounts ranging between 1% and 10% MPs in soil weight [24,25,45]. Our results fit and support the previous studies, confirming the hypothesis that, independently of their size, MPs might not induce oxidative stress in *E. foetida* under most environmental conditions. However, some studies showed the modulation of antioxidant enzymes and the onset of oxidative stress in earthworms exposed to MP abundances similar to those we administered in the present study. The discrepancy in findings on oxidative stress-related biomarkers can be explained by the duration of the exposure. Indeed, the studies highlighting effects on antioxidant enzymes generally exposed earthworms for 28 days, while in this study, the exposure lasted only 14 days. Thus, although all the studies confirmed MP ingestion, 14 days of exposure could not be enough for MPs to be incorporated in tissues and cause cellular effects. An additional source of the differences in oxidative stress-related responses can be identified in the portion of the earthworm body used for the analyses. In the present study, only the anterior part before the clitellum was used, while in previous studies, the analyses relied on the whole body. As the effects of MPs can depend on target tissue incorporation and damage, the selection of a portion or the whole body can contribute to variability in responses.

No effect at the individual level, in terms of gross growth rate and reproductive output, was noted in earthworms exposed for 14 days to both amounts of Px-MPs and APx-MPs (Figure 5). These results agreed with those obtained by previous studies performed on the same model species after exposure to different MPs, suggesting that the amount and size of MPs are the main determinants of toxic effects. For instance, growth inhibition was observed only after exposure to PS-MP amounts higher than 1% of soil weight [46], or to high concentrations (250 and 500 g/Kg soil weight) of PPC [30]. Moreover, because of their smaller size, larger specific surface area, higher adsorption capacity and probability of being ingested/retained in the digestive tract [25,38], small-sized MPs and nanoplastics (1 nm to <100 nm) reduced earthworm growth more than large-sized MPs [47,48,49]. Similarly, impairments of earthworm reproduction occurred only at high amounts of MPs [30] and depended on particle size and type of MPs. For instance, smaller nylon MPs induced a greater inhibition of earthworm reproduction than larger ones, while no effects were caused by PVC-MPs [50]. In contrast, no effects on cocoons [38] and juveniles [43] were found in *Lombricus terrestris* and *Eisenia andrei* earthworms, respectively, after exposure to low amounts of PE-MPs. Our results agreed with those reported in the current scientific literature, suggesting that the amount of MPs rather than the plastic type and the size represents the main determinant of adverse effects in earthworms. Indeed, a significant reduction in the number of cocoons and juveniles occurred after exposure to 53 g/Kg soil weight and 97 g/Kg soil weight, respectively, while no effect on survival was observed until exposure to an amount of 500 g/Kg soil weight [30].

## 5. Conclusions

Our study showed that MPs originating from naïve (Px-MPs) and additivated Plasmix-based materials (APx-MPs) were ingested by earthworms but were not able to negatively affect their health status. These data suggest that Plasmix-based materials might be considered as eco-safe at 0.1 and 1% Px-MPs in soil weight and 0.1 and 1% APx-MPs in soil weight. However, the lack of adverse effects could be due to the low amounts of MPs we administered, the short duration of the exposure, the selection of the part of the body used for analyses or the low sensitivity of the model species we used. Indeed, previous studies suggested that only the administration of MP amounts higher than those we administered induced toxic effects on earthworms. Thus, further studies administering higher MP amounts for longer exposure times should be helpful to confirm the negligible toxicity of these materials towards terrestrial organisms. Moreover, considering that MPs can enter aquatic ecosystems and easily interact with aquatic organisms, studies focused on the potential toxicity of MPs originating from Plasmix-based materials should be conducted to have an overview of the potential impact of these new materials on living organisms and to allow the creation of objects.

## Figures and Tables

**Table 1:** Heating program used for the GC-MS analysis.

State	Rate (°C/min)	Temperature (°C)	Hold Time (min)
Initial		45	2.0
Ramp 1	3	100	0.1
Ramp 2	5	135	0.1
Ramp 3	8	250	2.1

**Table 2:** Morphometric features (mean ± SEM) of Px-MPs and APx-MPs.

	Area	Perimeter	Diameter	Circularity
Px-MPs	34,937.10 ± 3583.03	589.24 ± 19.81	161.46 ± 5.67	0.74 ± 0.01
APx-MPs	248,081.10 ± 13,632.54	1849.19 ± 46.14	500.53 ± 12.21	0.75 ± 0.01

**Table 3:** Size distribution (%) of Px-MPs and APx-MPs administered to earthworms.

Class Size	<50 μm	50 < x <100 μm	100 < x < 250 μm	250 < x < 500 μm	500 < x < 750 μm	750 < x < 1000 μm	>1000 μm
Px-MPs	4.4	36.0	43.5	13.3	2.1	0.5	0.3
APx-MPs	0.2	0.1	9.3	50.2	26.1	9.5	4.5

**Table 4:** The name of the identified molecules, CAS number and averaged scaled peak area values (Awa) from Px-MPs and APx-MPs.

Retention Time (min)	Compound Name	CAS No.	Px-MPs Awa	APx-MPs Awa
2.1	3-(4-Isopropylphenyl)-1,1-dimethylurea	34123-59-6	36,800	41,844
2.2	2-Methoxy[1]benzothieno[2,3-C]quinolin-6(5H)-one	70453-75-7	62,880	66,726
4.9	Trimethylsilyl 2,6-bis[(trimethylsilyl)oxy]benzoate	3782-85-2	41,004	543,23
8.5	tetraHydro-2,5-dimethyl-2H-pyranmethanol	54004-46-5	26,983	27,774
11.5	Acetic Acid	64-19-7	980	1023
12.5	2-Ethyl-1-hexanol	104-76-7	5816	5778
13.2	Benzaldehyde	100-52-7	3749	5133
13.7	Propanoic acid	79-09-4	1733	1655
14.5	5-Methyl-2-furancarboxaldehyde	620-02-0	988	1278
16.5	Acetophenone	98-86-2	3456	4210
17.7	1-Chlorododecane	112-52-7	21,321	21,667
18.9	1,4-cicloottanedione	55794-45-1	3459	3899
21.4	Octadeca methyl cyclo nonasiloxane	556-71-8	4324	3806
23.4	1-Chlorotetradecane	2425-54-9	988	1023
24.9	Levoglucosenone	37112-31-5	4927	4478
25.8	Phenol	108-95-2	1514	1277
31.3	Caprolactam	105-60-2	36,030	24,102
40.8	(2-Phenylcyclobutyl) benzene	20071-09-4	2219	1519
42.1	Benzoic acid	65-85-0	2512	2822
47.8	Tetradecanoic acid	544-63-8	3002	2539
51.3	Hexadecanoic acid	57-10-3	37,900	10,211
56.1	Stearic acid	57-11-4	18,020	13,114
56.9	tert-Butylhydroquinone	1948-33-0	18,744	12,676
57.6	1,2-Benzenedicarboxylic acid	117-81-7	11,774	19,876
58.8	Oleic Acid	112-80-1	1408	1022

## Data Availability

Data will be shared upon request.
